# Improving and Comparing Probiotic Plate Count Methods by Analytical Procedure Lifecycle Management

**DOI:** 10.3389/fmicb.2021.693066

**Published:** 2021-07-12

**Authors:** M. L. Jane Weitzel, Christina S. Vegge, Marco Pane, Virginia S. Goldman, Binu Koshy, Cisse Hedegaard Porsby, Pierre Burguière, Jean L. Schoeni

**Affiliations:** ^1^Expert Committee on Measurement and Data Quality, US Pharmacopeial Convention, Rockville, MD, United States; ^2^Independent Consultant, Winnipeg, MB, Canada; ^3^Analytical Development, Bacthera, Hørsholm, Denmark; ^4^Probiotical Research Srl, Novara, Italy; ^5^Science Division, Department of Dietary Supplements and Herbal Medicines, US Pharmacopeial Convention, Rockville, MD, United States; ^6^Advanced Microbial Analytics (AMA) Research Solutions, Lyon, France; ^7^Eurofins Microbiology Laboratory, Madison, WI, United States

**Keywords:** probiotics, USP, colony-forming units, enumeration, analytical procedure lifecycle management, analytical target profile, target measurement uncertainty, methods comparison

## Abstract

Probiotics are live microorganisms that confer a health benefit to the host when administered in adequate amounts. This definition links probiotic efficacy to microbial viability. The current gold standard assay for probiotic potency is enumeration using classical microbiology plating-based procedures, yielding results in colony-forming units (CFU). One drawback to plating-based procedures is high variability due to intrinsic and extrinsic uncertainties. These uncertainties make comparison between analytical procedures challenging. In this article, we provide tools to reduce measurement uncertainty and strengthen the reliability of probiotic enumerations by using analytical procedure lifecycle management (APLM). APLM is a tool that uses a step-by-step process to define procedure performance based on the concept that the reportable value (final CFU result) must be fit for its intended use. Once the procedure performance is defined, the information gathered through APLM can be used to evaluate and compare procedures. Here, we discuss the theory behind applying APLM and give practical information about its application to CFU enumeration procedures for probiotics using a simulated example and data set. Data collected in a manufacturer’s development laboratory is included to support application of the concept. Implementation of APLM can lead to reduced variability by identifying specific factors (e.g., the dilution step) with significant impact on the variability and providing insights to procedural modifications that lead to process improvement. Understanding and control of the analytical procedure is improved by using these tools. The probiotics industry can confidently apply the information and analytical results generated to make decisions about processes and formulation, including overage requirements. One benefit of this approach is that companies can reduce overage costs. More reliable procedures for viable cell count determinations will improve the quality evaluation of probiotic products, and hence manufacturing procedures, while ensuring that products deliver clinically demonstrated beneficial doses.

## Introduction

Probiotics are “live microorganisms that, when administered in adequate amounts, confer a health benefit on the host” (Hill et al., 2014). Probiotic preparations must meet strict criteria related to quality, safety, and functionality (Vankerckhoven et al., 2008; Binda et al., 2020). A key quality criterion is that they contain accurately defined numbers of live cells as expressed on the product label. Hence, it is critical to accurately enumerate the population of live microbes in the preparation and express this information to the consumer on the product label. This presents a major analytical challenge for the probiotics industry as enumeration becomes paramount to assessing the quality of commercial probiotic products. There are numerous approaches to the measurement of probiotic cell viability including measurement of colony-forming units (CFU) by plating, flow cytometry, viability quantitative PCR, and droplet digital PCR (Hansen et al., 2018, 2020; Kumar and Ghosh, 2019). These methods or approaches measure different aspects of cell viability.

Most recognized standards such as those published by the International Standards Organization (ISO), International Dairy Federation (IDF), and United States Pharmacopeia (USP) use plate count procedures for bacterial enumeration of beneficial bacteria as well as contaminants (ISO, 2003, 2006, 2010; USP, 2013). The current standard in the probiotics industry is to measure probiotic potency using traditional microbiological plate count procedures, which fulfill growth requirements (i.e., nutrients, temperature, atmosphere). The benefits of plate counts are technical simplicity and ease of implementation. The challenges associated with plate count procedures are mainly related to laborious manual handling and variables within the procedure. Culture-based procedures generate counts with large total error [15–30% coefficient of variation (CV); Corry et al., 2007] and with varying degrees of intermediate precision and reproducibility. Limits ranging from 0.2 to 0.5 Log_10_ for the critical difference between two tests at the 95% confidence interval can be found in international standards and national guidelines. Additionally, no single plating procedure is applicable to all probiotic organisms, as there are considerable differences in growth requirements between bacterial species and strains as well as their manufacturing conditions (Davis, 2014). Therefore, a means to reduce variation is needed to obtain accurate CFU counts of probiotic products. Estimation of measurement uncertainty (*MU*) provides a means to assess and compare the overall variability of an analytical procedure carried out within a single laboratory or within different laboratories. Uncertainty and procedure variability associated with CFU enumeration of probiotic strains call for qualified procedures that can be applied for robust enumeration of culturable cells. The use of such procedures and the data generated provides more reliable quality metrics for the industry.

The United States Pharmacopeia (USP) is an organization known for creating quality standards for drugs, excipients, dietary supplements, and foods. These quality standards include monographs for probiotic ingredients, dietary supplements, and finished products which cover the identification, purity, assay, and contaminants. In response to the rapid and wide-spread growth of probiotic usage and subsequent increasing requests for probiotic monograph development, the USP formed a Probiotics Expert Panel (PEP).

Initially, probiotic monographs were developed for individual strains of a probiotic species and organizations submitting data included detailed CFU enumeration information that followed qualified analytical procedures. The number of CFU enumeration methods increased as monograph submissions for different strains within the same species increased. Most of these methods varied in parameters and qualification procedures, which underlined the need for tool(s) for qualifying and comparing different CFU enumeration procedures.

The approach for qualification and comparison of analytical procedures for live bacterial products, needs to accommodate the diversity of probiotic products, throughput of analyses, procedure uncertainty, and most importantly, must be accepted by manufacturers and regulators. Actions undertaken to understand analytical procedures will provide considerable opportunities for improving data quality as well as overall probiotics quality from commercial, regulatory, and consumer perspectives.

In this paper, analytical procedure lifecycle management (APLM; Martin et al., 2013) combined with tolerance interval (*TI*) calculations is used to compare analytical CFU enumeration procedures and provide a framework for implementation of this approach. Lifecycle management has been used for diverse applications, e.g., monitoring and improving chemicals, biologicals, drugs, immunoassays, information technology systems, biotechnological processes, and product marketing. However, these tools have not previously been applied to the evaluation of analytical procedures for live bacterial products.

Here, steps are detailed for developing APLM for CFU enumeration of probiotics. An in-depth APLM analysis, in the form of results for a simulated probiotic powder example using randomly generated data sets and statistical comparisons demonstrates the approach. The information used to generate the data is based on known variability in probiotic CFU measurements. The example identifies, defines, evaluates, and applies basic APLM principles to enumeration procedures and is followed by statistical analysis using *TI* as described in “USP <1210> Statistical Tools for Procedure Validation” (USP, 2018). To further support the value of this approach, real-life data for a *Lactobacillus acidophilus* powder is included. Combined, APLM and *TI* calculations characterize procedure performance, furnish a basis for comparing procedures, and provide tools that the probiotics industry can use to improve the reliability of their decision-making data and increase product consistency.

## Materials and Methods

The theory and principles of APLM are presented. Consecutively and step-by-step, the theory and principles of APLM are applied to a general illustrative example, which demonstrates and further elaborates the potential of this approach. New terms introduced by APLM and others relevant to this paper are defined in the glossary (Supplementary Appendix 1). The uncertainty classifications from ISO 19036:2019, Microbiology of the Food Chain – Estimation of Measurement of Uncertainty for Quantitative Determinations, are used for some statistical calculations (ISO, 2019).

## Details for the Example

The analytical procedure used for the general example determines culturable cells of *Lactobacillus* spp. as CFU/g in a powder. Data were randomly generated using the MS EXCEL (2016) to demonstrate performance of sample enumeration via agar plating. The function NORMINV was used to return numbers that were normally distributed around a mean, altered by a standard deviation and by a probability factor. The provided means, standard deviations, and probability applied for the example are based on empiric knowledge, expertise, and experience. In this example, the manufacturer states on the certificate of analysis (CoA) that the powder contains ≥91.67 billion CFU *Lactobacillus* spp./g or 10.962 Log_10_ CFU/g. In the example, the CoA claim is also referred to as the lower limit. Manufacturing overage was set at 0.500 Log_10_ above the planned CoA claim to ensure potency throughout product shelf life. The manufacturer’s internal release specification, which accounts for overage, is 11.462 Log_10_ CFU/g. The laboratory plans to plate dilutions that will cover two Log_10_ CFU/g above and below internal release specification, i.e., the procedure will be applicable for CFU enumeration of 9.462–13.462 Log_10_ CFU *Lactobacillus* spp./g powder. Selected dilutions from each test sample will be plated in triplicate. Although various counting ranges exist, for this analysis the laboratory uses 25–250 colonies per plate.

### Rounding and Significant Figures

Internal policies on rounding and significant figures may be followed. Generally, final uncertainty is given using two significant figures. The reportable value should be rounded to be consistent with the uncertainty. For this example, Log_10_ values are shown to three decimal points. In actual calculations all digits are used. This may result in minor discrepancies in values. The specification values, which are most often reported as arithmetic numbers, show two decimal points when referring to the CoA.

## Analytical Procedure Lifecycle Management (APLM)

Good manufacturing practices (GMP) require analytical procedures used for potency analysis of probiotics demonstrate fitness for intended use. APLM is a holistic model that encompasses the traditional approaches to procedure development, qualification, verification, and transfer rather than viewing these concepts as separate entities. Moreover, an analytical control strategy (ACS) is applied to ensure the analytical procedure remains in a stage of control throughout the lifecycle.

APLM is based on the reportable value, in this case the CFU concentration for CFU enumeration analytical procedures, being fit for its intended use. Hence, the intended use must be clearly defined and understood. In APLM, the intended use of an analytical, quantitative procedure is defined by developing an analytical target profile (ATP) as defined in USP PF 46(5) (USP, 2020), “Analytical Procedure Life Cycle”:

“The ATP is a prospective description of the desired performance of an analytical procedure that is used to measure a quality attribute, and it defines the required quality of the reportable value produced by the procedure.”

The ATP provides the information needed to set procedure qualification criteria. As such, it can also provide criteria for comparing analytical procedures. The three steps used to develop an ATP will be discussed in detail:

1. Develop the measurand which describes what is being measured.
2. Develop the decision rule which describes the maximum acceptable measurement uncertainty (*MU*) or target measurement of uncertainty (*TMU*) and acceptable probability of being wrong.
3. Develop the ATP.

**FIGURE 1:**
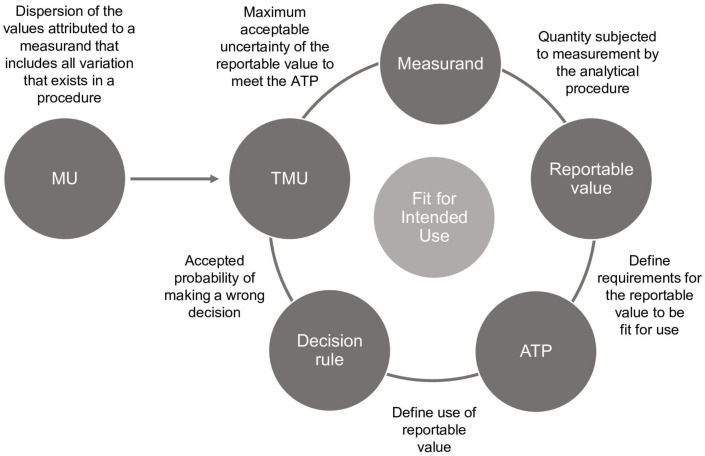
The ATP and its components are related and interactive. If any component changes, another component may need to change also. MU, measurement uncertainty; TMU, target measurement uncertainty; ATP, analytical target profile.

As seen in Figure 1, ATP and its components (measurand, decision rule, and *TMU*) are interactive. Therefore, the fitness for intended use needs to be evaluated and adjusted according to performance throughout the lifecycle of the procedure.

### The Key Components in APLM and How to Use Them

#### Step 1 Develop the Measurand

The measurand, as defined in ISO 19036:2019 (ISO, 2019), is the quantity subjected to measurement. The measurand is developed using information from prior knowledge, ingredient manufacturing, product formulation, and analytical development. In this step, a complete statement of the item being analyzed is developed. It may include, but is not limited to, details such as the probiotic microorganism(s), matrix, product form, units of measure, possible contaminants, and/or impurities. Moreover, the entity for which the decision will be made (e.g., the lot or batch of bulk in the warehouse), and the entity used to make the decision (i.e., a representative sample, a laboratory sample, a composite, or single grab), are also defined.

The advantage of defining the measurand is that it provides a mechanism for communicating to all parties exactly what is being measured. Table 1, which includes a series of questions and example answers pertinent to probiotic ingredient powders, was compiled as an aid to developing measurand statements specifically for probiotics.

**TABLE 1:** Questions useful for identifying the information needed to define the measurand.

Question(s)	Answer and/or guidance for a specific product
What is the analyte? What is being detected? What is being counted?	The analyte is the entity measured by the analytical procedure. The analyte is culturable cells enumerated as colony forming units (CFU).
What is the matrix? Are there excipients? Stabilizers?	Matrix components are generally cryoprotectants, excipients, bulking agents for powder flow, etc.
Are there possible contaminants in the matrix?	Non-microbial contaminants: Carryover from fermentation media, leachables, and extractables from production systems. Microbial contaminants (both live and dead), remnants of cell-walls, cross-contaminants (from other strains produced in the same facility), and environmental contaminants.
Will the term “pure” be used to describe the ingredient?	Probiotic ingredients are often described as “pure” powders. The term “pure” is controversial but useful. The discipline of defining a measurand requires that the meaning of the controversial term, pure or purity, be defined if used. The probiotic ingredient (freeze dried cells) is usually a pure powder that may contain cryoprotectant and carryover of fermentation media. It does not contain excipients as do final formulated product.
Matrix: other components.	Is the probiotic ingredient a pure powder? Is the probiotic ingredient definition, above, used to describe the term pure? Is the probiotic ingredient in a solution or suspension? Include the solvent or suspension liquid in the measurand definition. Usually, there are no solvents in a freeze-dried product.
What is the decision unit (also known as parent body)? For what entity will the decision be made?	Options to consider for the decision unit: Laboratory sample, a batch of probiotic ingredient, a product lot. Composite sample or a single grab sample. Randomly selected from a bulk-capsule or finished capsule lot. The sample taken from the beginning, middle, or end of the batch, or at all three time points. The form of the sample is a bulk ingredient, formulated blend, capsules, sachets. R&D may conduct a study during process development to ensure the sample is representative, and that the uncertainty contribution from sampling is not of practical importance.
What is the physical form of the decision unit?	Powder, solution, etc. Describe the form.
Define the units for the quantity.	For example, the unit can be CFU/g or CFU/mL.

The information in the following two points is not needed to define the measurand; but is needed to complete the ATP. It is convenient to collect this information along with details for defining the measurand.	
What is the concentration range of test results that should be reported by the analytical procedure?	The laboratory may extend that range to include concentrations for potentially OOS values. This information is usually provided by the development team.
What is the counting range?	There are different standards for the counting range. The counting range depends on the size of the Petri dish, the applied agar, the probiotic strain, etc. It is up to the manufacturer to assess the counting range and the linearity for a specific ingredient and/or product with the applied CFU method.

*The answer for each question is either information applicable to the analytical probiotic enumeration procedure or guidance for a company’s specific product. These questions address both the probiotic ingredient and finished product.*

The initial definition of the measurand can be supplied by the department responsible for developing the probiotic product, often Research and Development. As a product development project advances, changes may be made to the development process and/or the formulation. For example, a new excipient may be added to the matrix. This change will trigger a revision to the definition of the measurand and everyone involved in developing the product and/or the relevant analytical procedures will be notified so the analytical procedure can be evaluated and adapted as needed.

##### Questions and Answers to Develop the Measurand for the Example Lactobacillus spp. Powder

Table 1 is presented as a tool for gathering relevant information used to define the measurand. The reader could copy Table 1 and use, fill in, or adapt the responses. Information gathered for the *Lactobacillus* spp. example follows:

• The analyte consists of culturable *Lactobacillus* spp. cells enumerated as CFU.
• The matrix is cryoprotectant. The cells are freeze-dried.
• The product is not manufactured with wheat, gluten, soy, milk, egg, fish, shellfish, or tree nut ingredients. It is produced in a GMP facility that processes multiple probiotic strains and other ingredients containing these allergens. It is expected that cells from other probiotic strain and allergens, if present, are in very low concentrations and do not impact the measurement.
• The term pure or purity is not used.
• The decision unit is the laboratory sample. During product development, it was established that the laboratory sample was representative of the lot of probiotic powder.
• The physical form is a powder.
• The unit for quantity is the concentration CFU/g. The laboratory decided to report results as CFU/g on CoA, while using Log_10_ transformed data for conducting statistical analyses and trending.

##### Measurand for the Example Lactobacillus spp.

Culturable cells (live cells freeze-dried) of *Lactobacillus* spp., CFU/g, in powder with cryoprotectant.

#### Step 2 Develop the Decision Rule

The decision rule defines the fitness requirements for an analytical procedure in the context of using the reportable value. It describes how measurement uncertainty will be considered when deciding whether to accept or reject a product according to its specification and the result of a measurement. In other words, the decision rule is a prescription for the acceptance or rejection of a probiotic product based on the reportable value, its uncertainty, and the specification limit or limits, considering the acceptable level of the probability of making a wrong decision. Documentation of the decision rule is critical to ensuring clarity of these requirements.

**FIGURE 2:**
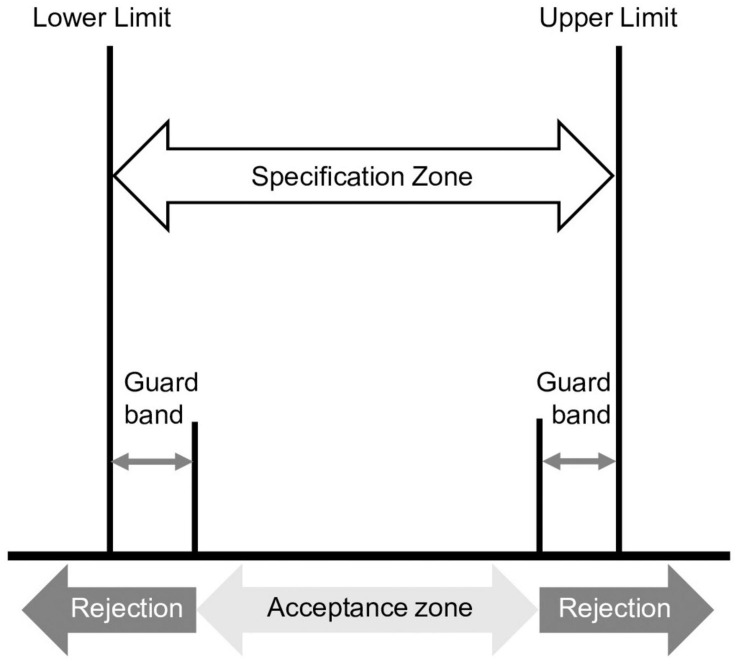
The elements of a decision rule illustrated for a specification with upper and lower limits. A guard band is used to control the probability of making a wrong decision. In this case, the acceptance zone is smaller than the specification zone.

Four components are included in the decision rule: (i) product specification (CoA claim) often with guard bands or coverage factors to set decision limits; (ii) the acceptable probability for making an incorrect decision, e.g., erroneously accepting a lot that does not meet specifications or rejecting a false out-of-specification (OOS) lot; (iii) a defined reportable value; and (iv) the standard uncertainty (*u*) associated with the reportable value. The decision rule can be formulated using information from sources external to the laboratory such as the customers, stakeholders, decision makers, and risk managers. Figure 2 shows the elements of a decision rule for a specification with lower and upper limits. Use of an upper limit may depend on the country and regulatory classification of the final product. Overdosing or adding overage is a common practice within the industry, used to ensure and maintain label claims. It is expected that overage has been added to a level that will maintain concentrations greater than or equal to the CoA claim specification throughout the shelf life of the product.

**FIGURE 3:**
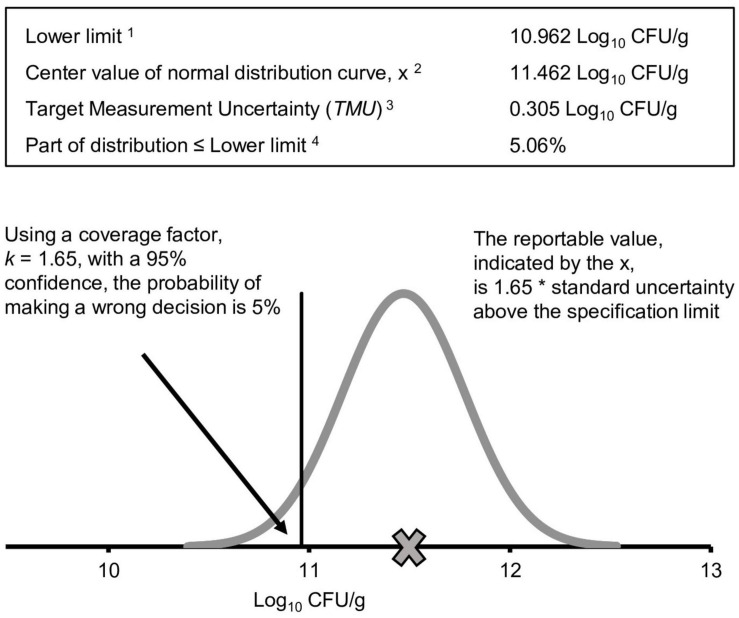
Illustration and identification of the target measurement uncertainty (*TMU*). When the reportable value, shown by the **x**, is 1.65 × standard uncertainty above the lower limit, the probability of being wrong is 5%. ^1^Lower specification limit from the example *Lactobacillus* spp. ^2^X, the reportable value, is also the mean or mid-point of the distribution. ^3^For determining the *TMU*, the MS EXCEL formula, = NORM.DIST, can be used. For a lower limit, the formula is = NORM.DIST(lower limit, X, TMU, TRUE). In this example, the reportable value must be above the label claim to release the product. Therefore, lower limits are designated by label claims. The value of the *TMU* is varied until the formula matches the desired probability of being wrong. ^4^Probability of being wrong defined in the decision rule.

The decision rule defines the use of the reportable value and provides the acceptable probabilities for making a wrong decision with the reportable value. These acceptable probabilities are needed to set the *TMU*, defined in VIM (ISO/IEC, 2015) as “measurement uncertainty specified as an upper limit and decided on the basis of intended use of measurement results.” The TMU for an analytical procedure must be consistent with the decision rule and the values specified within. Thus, the decision rule can provide an understanding of the maximum variability or *TMU* (see Figure 3 for more information) that can be associated with a reportable value to allow the result to remain fit for its intended use. *TMU*, which considers intended use of the measurement, can become part of the ATP.

When there is a defined limit for the measurand, typically in legislation or a technical specification, there may also be guidance about the acceptable magnitude of the uncertainty. For probiotics, the potency specification is usually a minimum limit. When reference documents for the specification limit of the measurand do not include *TMU*, this requirement needs to be defined in another way. Empirical knowledge, data, and risk management can contribute to the determination of *TMU*.

The process for calculating *TMU* is described later in this paper. For detailed discussions of decision rules and *TMU*, readers are directed to the following references: USP stimuli article, “Fitness for Use: Decision Rules and Target Measurement Uncertainty” (Burgess et al., 2016); Guidelines by Ellison and Williams (2012) and American Society of Mechanical Engineers (2019); and Weitzel and Johnson (2012).

##### Mathematical Considerations Pertaining to the Decision Rule

*Log_10_ (CFU) and Normal Distributions*. Probiotic CFU counts generally follow a Log_10_-normal distribution. Log_10_ transformed data is used to calculate the *TMU*. Care needs to be taken when using and interpreting Log_10_ transformed data, e.g., Log_10_ numbers cannot be added or subtracted to calculate the difference between the numbers. More information on the Log_10_ transformation is available from the World Health Organization (WHO) guide *Statistical Aspects of Microbiological Criteria Related to Foods* (FAO/WHO, 2016).

If counts do not follow a Log_10_-normal distribution, there are other techniques to use, but these are beyond the scope of this paper.

*Expanded Uncertainty (U) and Coverage Factors (k)*. The expanded measurement uncertainty, or just expanded uncertainty (*U)*, is an interval around a measurement result that is expected to encompass a large fraction of the distributed values that could reasonably be attributed to the measurand. The fraction may be regarded as the coverage probability or level of confidence of the interval (ISO, 2019). Guides on uncertainty recommend that laboratories report the expanded uncertainty because it provides an interval within which the true value is believed to lie with a higher level of confidence than for a standard uncertainty. Expanded uncertainty is calculated from the standard uncertainty (*u*) and a coverage factor (k_*pr*_): *U* = *u × k*_*pr*_ (Supplementary Appendix 2). The coverage factor is chosen based on the acceptable probability of making a wrong decision (*pr*). The coverage factor is like the *Z* factor for a standard normal distribution. For a two-tailed distribution, at the 95% level of confidence, *k_*pr*_* = 1.96, but the estimate 2 is often used in calculations.

An example to assess compliance with a lower limit only (a one-tailed distribution) is illustrated in Figure 3. To have 95% confidence that a reportable value is above the specification (company has decided that the acceptable probability for making a wrong decision is 5%), the standard uncertainty for the value is multiplied by a coverage factor, *k_*pr*_.* The *k*_*pr*_ value is obtained from a *Z* factor table for confidence levels of one-tailed normal distributions (Devore and Beck, 2011). The coverage factor for the example is *k*_*pr*_ = 1.65. Under the condition of 95% confidence (probability of making wrong decision is 5%), the result must be 1.65 × standard uncertainty (*u*) above the lower specification limit for the reportable value to comply with the specification of 95% confidence. Since the expanded uncertainty (*U*) is calculated using *k*_*pr*_, *TMU* can be calculated by dividing the desired expanded uncertainty range by *k*_*pr*_:

$$TMU=U/k_{pr}\left(\mathrm{Eq}.1,\text{Supplementary Appendix 2}\right)$$

##### Measurement Uncertainty

VIM (ISO/IEC, 2015) defines measurement uncertainty as a “non-negative parameter characterizing the dispersion of the quantity values being attributed to a measurand, based on the information used.” It describes the range in which the true value is expected to be. Measurement uncertainty includes all random variation that exists in each step of the analytical procedure.

A detailed description of measurement uncertainty (*MU*) is provided in the USP 44(1) stimuli article, “Measurement Uncertainty for the Pharmaceutical Industry” (Weitzel et al., 2018). Guides on how to evaluate measurement uncertainty are provided by Eurachem (Ellison and Williams, 2012), ISO 19036:2019 (ISO, 2019), and MIKES (Niemelä, 2003).

##### Wording of Decision Rules

The decision rule can be written in different ways. A regulatory agency may not have specific information about a probiotic product, so it would write a general decision rule. The regulatory requirement states that a product is acceptable if the reportable value is within the specification range. In the United States, the specification range for a probiotic product is the label claim. When a value at the lower limit of specification (label claim) is obtained, the product is acceptable. The normal distribution curve (representing the expanded uncertainty or the range in which the true value may lie) is then centered over the limit. Hence, half of the normal curve is below the limit and half of the curve is above the limit. This means that there is a 50% probability the true value is below the limit and a 50% probability the true value is above the limit. A manufacturing company can apply the regulatory decision rule or write a more conservative rule based on the company risk profile and inclusion of specific information for its product.

The regulatory decision rule could be:

The decision unit, which is the batch of powder (culturable cells or spores, freeze- or spray-dried) will be considered compliant with the specification (100% label claim) if the probability of being wrong is ≤50%. Otherwise, it will be considered non-compliant.

A general format for company decision rules could be:

The decision unit, which is the batch of powder (culturable cells or spores, freeze- or spray-dried) will be considered compliant with the specification of SPEC if the measurement uncertainty is less than the *TMU* and probability of being wrong is ≤5%. Otherwise, it will be considered non-compliant.

SPEC means the manufacturer’s specification and *TMU* is the manufacturer’s *TMU* value. The company includes its values for SPEC and *TMU* in its decision rule.

##### Developing the Decision Rule for the Example Lactobacillus spp. Powder

First, the components included in the rule were defined.

1. The product specification often with guard band(s) is used to set decision limits. The product specification becomes the acceptance zone.
  a. The specification is *Lactobacillus* spp. concentration ≥91.67 billion CFU/g which is 10.962 Log_10_ CFU/g.
  b. The company includes an overage of 0.500 log_10_ CFU/g. For this powder, the manufacturing variability has been well characterized and is much less than 0.500 Log_10_ CFU/g. This means the laboratory samples will have values close to 11.462 Log_10_ CFU/g.
  c. In this example, the label claim is applied as the specification. A more cautious approach would be to apply a higher release specification to compensate for loss of culturability throughout shelf life
2. The acceptable probability for making an incorrect decision, e.g., erroneously accepting a lot that does not meet specifications or rejecting a false OOS lot.
  a. The decision makers provide the acceptable probability of being wrong and releasing a lot that is below specification as 5%.
  i. The acceptable probability of being wrong can be any percentage the company chooses and is willing to accept. In many industries and in this example 5% is selected.
  b. In this example, it is not likely that the probability of erroneously accepting a lot that is manufactured below CoA claim will be significant because there is relatively low variation in the manufacturing process and the overage ensures that the *Lactobacillus* spp. concentration will be above the CoA claim.
3. A defined reportable value.
  a. The reportable value is that which is obtained for each lot in routine testing.
4. The standard uncertainty (*u*) associated with the reportable value.
  a. This uncertainty is the *TMU* to meet the requirement of the acceptable probability of being wrong, which is 5% for this example.

After defining the components of the decision rule, *TMU* is calculated. *TMU* can be determined using the equation in Supplementary Appendix 2, using available calculators, or by creating a MS EXCEL spreadsheet using the NORMDIST formula as shown in Figure 3. In the example *Lactobacillus* spp., *TMU* = 0.305 Log_10_ CFU/g.

The decision rule for the example *Lactobacillus* spp.:

The laboratory sample, taken from the batch of *Lactobacillus* spp. probiotic powder (culturable cells, freeze-dried) will be considered compliant with the specification of 10.962 Log_10_ CFU/g if the reportable value is ≥10.962 Log_10_ CFU/g, the *MU* is <0.305 Log_10_ CFU/g, and the probability of being wrong is ≤5%. Otherwise, it will be considered non-compliant.

#### Step 3 Develop the ATP

The ATP is essential to the APLM. It stipulates the required quality of the reportable value and provides clear, pre-defined objectives for performance of the analytical procedure. Components of the ATP should express the definition of the measurand, as well as the requirements specified in the decision rule where performance of the procedure and external factors have been considered. For further information on ATP development for analytical procedures that are in accordance with USP guidance refer to Martin et al. (2017); Barnett et al. (2016), and USP PF 46(5), “<1220> Analytical Procedure Lifecycle” (USP, 2020).

The wording of the ATP can follow the format provided in “Proposed New USP General Chapter: The Analytical Procedure Lifecycle <1220>” (Martin et al., 2017):

“The procedure must be able to quantify [analyte] in the [description of test article] in the presence of [x, y, z] so that the reportable values fall within a *TMU* of ± C%. The probability of being wrong must be less than w%.”

The ATP provides and informs the acceptance criteria for analytical procedure qualification. Moreover, the ATP can be applied for performance comparison of different analytical procedures.

##### Analytical Target Profile (ATP) for the Example Lactobacillus spp.

Using the information from the measurand and the decision rule, the ATP for the example *Lactobacillus* spp. was written following the proposed USP format (Martin et al., 2017).

The procedure must be able to enumerate the *Lactobacillus* spp. culturable cell count in CFU/g of powder with cryoprotectant, formulated to 11.462 Log_10_ CFU/g, so the reportable values fall below a *TMU* = 0.305 Log_10_ CFU/g (i.e., the *TMU* associated with the reportable value is <0.305) and the probability of being wrong is ≤5%. The plating range used by the laboratory will cover 9.462–13.462 Log_10_ CFU/g, two Log_10_ above and below the internal release specification.

### Analytical Procedure Development and Qualification

The ATP is used to guide analytical procedure development and qualification. This paper does not discuss these topics in detail; but does provide some experimental approaches useful for these activities.

Qualification activities consist of designing and conducting experiments that will demonstrate the procedure is performing as required and focus on evaluating the measurement uncertainty (ISO, 2019; Martin et al., 2017). Procedure variables and parameters that carry uncertainty need to be included in qualification experiments. Variables that carry uncertainty can be determined through a risk analysis of the analytical procedure. Risk analysis is discussed in detail under the section Quality Risk Management (QRM).

#### Bias

Bias (or accuracy) is not included for microbiological CFU enumeration procedures because, currently, there are limited CFU or proliferation-based reference standards with assigned, certified, or reference values available. Also, for experiments it is difficult to create test samples that have the same value because the analyte (culturable cells), changes with time. For these reasons, the experiments focus on determining the precisions: repeatability and intermediate precision.

#### ANOVA Experiments

There are many ways to determine the repeatability and intermediate precision. In our process, analysis of variance (ANOVA) experimental design is used to determine repeatability and intermediate precision. The actual performance of an analytical procedure in routine use is expressed by varying the conditions, including those identified through risk analysis (section “Risk Analysis for the Example *Lactobacillus* spp.”), used in the experimental runs (replicates) of the ANOVA design. For example, multiple lots of agar media or different operators can be used. The experimental conditions should capture as many possible scenarios for operating the analytical procedure as is practical.

The impact of variables is evaluated in ANOVA experiments. It is acceptable to group variables into experimental “conditions” to streamline work. However, using conditions or grouping variables does not allow the uncertainty for each variable to be estimated. The conditions simulate scenarios that could be seen during routine use of the procedure. The ANOVA design for the example *Lactobacillus* spp. is included in section “ANOVA Design for the Example *Lactobacillus* spp.”

For qualification of the analytical procedure, the ANOVA statistical tool is applied to the gathered data and ANOVA analysis of the values provides the uncertainty for the specified procedure and the probability of being wrong. Values determined through qualification activities are compared to those stated in the ATP. If the values determined through qualification are less than or equal to those stated in the ATP, the procedure is performing as required.

##### Precision From Replicating Steps in an Analytical Procedure

The uncertainty contributed from steps that are replicated in an analytical procedure can be estimated. A common replication is to duplicate steps. For example, multiple representative test portions can be taken from a sample sent to the laboratory. Here, assume that one test portion is used to prepare an initial suspension. The initial suspension is used to create two series of dilutions (technical replicates). The act of creating two series of dilutions, duplicates the step in the analytical procedure. Two technical replications (the dilution series) made from one test portion now exist. The technical replicates results can be analyzed to estimate the uncertainty created at the step in which the dilution series are prepared. A description of the use of duplicate or technical replicates is provided in Weitzel and Johnson (2013).

**FIGURE 4:**
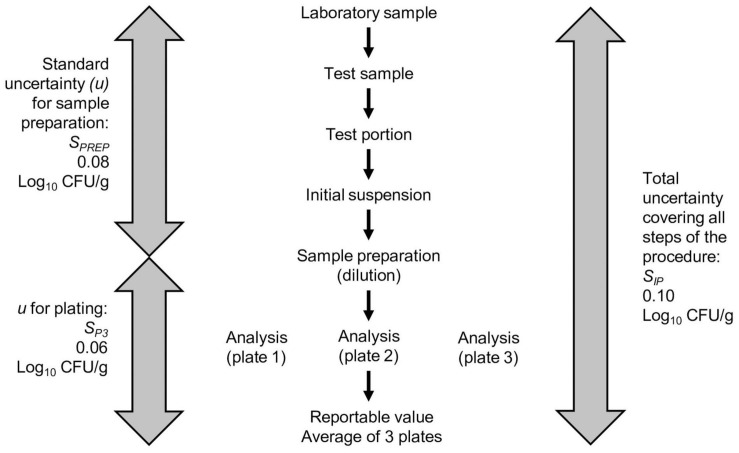
Flow chart of the *Lactobacillus* spp. enumeration procedure. For each analysis, three plate counts are generated. The standard deviations for the plate count (*S*_*p3*_) can be calculated from the triplicate plate count data to provide an estimate of the uncertainty from the plating and counting steps. The variance of the plate count can be subtracted from the variance covering the entire procedure to estimate the variance of the sample preparation (*S*_*PREP*_). The values shown are for the example *Lactobacillus* spp.

Another example of replication is seen in the use of triplicate plating for the CFU procedure in the example *Lactobacillus* spp. (Figure 4). One test portion of the laboratory sample is used to produce the initial suspension. The initial suspension is serially diluted, and three plates (diluted sample + agar medium) are generated from specified dilutions. Each plate is a replicate. The calculation of the uncertainty for the plating step from triplicate plate counts is demonstrated later in this article.

#### Quality Risk Management (QRM)

A cornerstone in the APLM approach is an analytical control strategy based on the process presented in the USP stimuli article, “Analytical Control Strategy” (Kovacs et al., 2016). The reader is referred to that paper for the explanation of the QRM theory and process. Briefly, QRM is a tool to obtain improved understanding of the link between procedure variables and the accuracy and precision of a reportable value, as well as the interdependencies of the different variables. The QRM process includes four major parts:

1. Risk assessment (comprised of the following three steps)
  a. Risk Identification
  b. Risk Analysis
  c. Risk Evaluation
2. Risk Control
3. Risk Communication
4. Risk Review

QRM identifies risks that need to be included in the evaluation of measurement uncertainty. These risks become the variables included in the ANOVA experiments to evaluate the intermediate precision, i.e., the intra-laboratory reproducibility (ISO, 2019).

Of specific interest for analytical procedures enumerating CFU and the comparison of such procedures, is identification of variables and potential risks involved with conducting an analytical procedure using plate count techniques producing CFU/g or CFU/mL as the reportable value. It is valuable to identify variables within the analytical procedure that may require control. Risk control may result in mitigation or understanding and acceptance of the risk without mitigation. Controlling risks has the potential to reduce uncertainty. These aspects are addressed in part 1 (risk assessment) and part 2 (risk control) of the QRM process.

Risk assessment starts with the risk (hazard) identification. This is when the question, “What might go wrong?” is asked. The laboratory identifies risks applicable to their specific analytical procedure. To gain additional insight into the enumeration procedure, the potential risks can be categorized as technical, matrix, or distributional as per ISO 19036:2019 (ISO, 2019). Technical uncertainty may also be called operational uncertainty. It is associated with the technical steps of the analytical procedure and covers items such as sampling, mixing, diluting, plating, and counting. Matrix uncertainty is related to how well the laboratory sample behaves when mixed, causing larger variability between test portions. Distributional uncertainty is intrinsic. It is an unavoidable variation associated with the distribution of the microorganisms in the sample, initial suspension, and subsequent dilutions.

##### Risk Analysis for the Example Lactobacillus spp

A risk analysis for the example *Lactobacillus* spp. is found in Supplementary Appendix 3. It includes a comprehensive list of potential risks that can serve as guidance for preparation of risk analyses associated with other probiotic enumeration analytical procedures. Risk control requires review of the potential hazards/risks associated with the analytical procedure and evaluation of the individual risks so that a mitigation strategy can be developed or to allow acknowledgment and acceptance of the risk without mitigation. Prior knowledge and experimental studies can be applied in making such decisions. It should be noted that in the work of this manuscript, microbiologists and probiotics enumeration specialists created both the extensive list of hazards/risks and the examples of mitigation strategies. However, neither the list of hazards/risks nor mitigations presented in Supplementary Appendix 3 are considered definitive. Each laboratory must identify the risks applicable to its procedure, evaluate them, and design appropriate mitigation strategies.

Theoretically, every step in the analytical procedure, from sampling to the final reportable value, has the potential to contribute to the *MU* of the reportable value. There may be one or more variables in each step. Strategies for the analytical procedure controls can be designed to reduce input variation or to adjust for input variation to reduce its impact on the output, or a combination of both approaches. The systematic approach to risk management ensures that the performance of the analytical procedure can be explained logically and/or scientifically as a function of procedure parameters and inputs and is most effective when supported by solid knowledge base. Sources of knowledge include prior knowledge (public domain or internally documented), expertise (education and experience), experience with similar applications, and product or process specific knowledge developed and/or acquired with each application as it becomes available.

Some risks are related and thus may require related mitigation strategies. For example, a counting error could have many sources. Discussions of relationships are included in Supplementary Appendix 3. Other risks can be handled by complying with GMP. Mitigation by GMP follows several assumptions:

• The analytical procedure is used in a GMP laboratory.
• The equipment is properly qualified.
• Calibration and preventive maintenance programs are in place.
• Analysts are trained and competent.
• Instructions, such as Standard Operating Procedures (SOP), analytical procedure descriptions, work instructions, etc. are in place. These must be in place before any experiments are conducted.
• There are control programs for media and reagents.

Examples where GMP compliance could be used as the mitigation strategy include: (i) The potential risk of an incubator exceeding its maximum load. If the incubator qualification demonstrates a maximum load for the incubator to maintain the temperature specification, then a GMP compliant laboratory will mitigate the risk of overloading by stipulating the maximum load in their SOP, analytical procedures, and/or policies. (ii) Risk of pipetting errors. Pipettors must be qualified and calibrated. (iii) Pipetting technique, which can cause a huge variance in results. Under GMP requirements, the laboratory should provide adequate instruction and training to ensure the analysts are competent and pipet consistently. When transferring analytical procedures, laboratories should consider and compare their pipetting techniques. The detailed instructions from the analytical control strategy assist with this comparison.

#### Analytical Procedure Qualification Activities for the Example *Lactobacillus* spp.

The purpose of analytical procedure qualification is to demonstrate that procedure performance meets the requirements outlined in the ATP. This is tested by an experimental design using variables determined to be significant through risk analysis of the procedure. The decision rule and ATP provide the goals and acceptance criteria for the qualification activities.

From the risk analysis of example *Lactobacillus* spp. (Supplementary Appendix 3), uncertainty components were identified as variables that should be included in the ANOVA experiments (Table 2). Most risks for this CFU enumeration procedure fell into the ISO category: technical uncertainty (ISO, 2019). The laboratory varies as many risks as practical to mimic routine experimental conditions.

**TABLE 2:** Uncertainty components for the simulated procedure qualification for the example *Lactobacillus* spp.

Uncertainty component	Condition			
	1	2	3	4
Days	A	B	C	D
Analyst	A	B	A	C
Lot of plating medium	1	2	1	2
Lot of suspension/rehydration medium	2	2	1	1
Lot of dilution buffer	1	2	3	4
Disposable serological pipettes	Lot 1	Lot 2	Lot 1	Lot 3
Pipettors with tips	Set A	Set B	Set A	Set C
pH meter	A	B	A	B
Analytical balance	1	2	2	1
Autoclave	1	2	3	2
Agar tempering water bath	2	1	1	2
Incubator	2	3	1	5

*The components were identified through the risk analysis, and the individual variation during the ANOVA experiment are shown for each condition.*

##### ANOVA Design for the Example Lactobacillus spp.

For illustration of a procedure qualification experimental design, simulated data were generated for the example *Lactobacillus* spp. The data represents the impact of conducting the procedure while varying uncertainty components.

For this qualification, the experimental design included:

• Four conditions with variable uncertainty components.
  ◦ The conditions cover variables encountered by plating to obtain a reportable value of 9.462–13.462 Log_10_ CFU/g powder.
• Ten replicates per condition.
  ◦ The entire analytical procedure is performed on each replicate.
  ◦ Each replicate represents one test portion of the laboratory sample.
  ◦ A single dilution series is conducted per replicate.
  ◦ Dilutions are plated in triplicate.
  ◦ The reportable value is the mean value of three plates. Plates counts must fall within the specified counting range, e.g., 25–250 CFU/plate.

The standard deviation, variance, and average for each condition is calculated, followed by calculation of pooled standard deviation. For this ANOVA, the between condition variance is not considered because the true or reference values for each condition are not known and cannot be controlled. An overview of the experimental design and ANOVA results are compiled in Table 3.

**TABLE 3:** The ANOVA experimental design and data for procedure qualification of the example, *Lactobacillus* spp.

Replicate	Counts in Log_10_ CFU/g															
	Condition 1				Condition 2				Condition 3				Condition 4			
	1	2	3	Average	1	2	3	Average	1	2	3	Average	1	2	3	Average
1	11.336	11.478	11.424	11.416	11.236	11.443	11.311	11.330	11.335	11.575	11.234	11.381	11.162	11.326	11.211	11.233
2	11.146	11.312	11.485	11.404	11.442	11.357	11.224	11.341	11.531	11.606	11.584	11.574	10.964	10.996	10.959	10.973
3	11.506	11.688	11.583	11.592	11.466	11.274	11.348	11.356	11.418	11.373	11.386	11.392	11.169	10.946	10.945	11.020
4	11.324	11.363	11.178	11.288	11.167	11.297	11.295	11.253	11.506	11.275	11.322	11.368	10.929	11.018	11.112	11.020
5	11.397	11.519	11.358	11.425	11.424	11.267	11.416	11.369	11.351	11.315	11.282	11.316	11.206	10.986	11.093	11.095
6	11.511	11.639	11.565	11.572	11.416	11.272	11.439	11.376	11.439	11.460	11.695	11.531	10.962	10.815	10.798	10.858
7	11.436	11.510	11.503	11.483	11.511	11.338	11.446	11.432	11.446	11.546	11.441	11.478	11.154	11.290	11.071	11.172
8	11.551	11.700	11.486	11.579	11.193	11.203	11.366	11.254	11.413	11.409	11.389	11.404	11.047	11.191	11.081	11.106
9	11.429	11.607	11.521	11.519	11.283	11.276	11.265	11.275	11.334	11.563	11.018	11.305	10.870	11.005	10.819	10.898
10	11.733	11.712	11.462	11.636	11.258	10.997	11.156	11.137	11.407	11.201	11.486	11.365	10.999	11.074	11.127	11.067
Std. Dev. (*S*_*C*_)				0.1080				0.0841				0.0888				0.1160
Variance (*S_*C*_^2^*)				0.0117				0.0071				0.0079				0.0134
Average (*C*)				11.491				11.312				11.411				11.044
Intermediate precision = Pooled Std. Dev. (*S*_*IP*_)				0.1001												
Std. Dev. for single plate count (*S*_*P1*_)				0.1033												
SEM for average of three plate counts (*S*_*P3*_)				0.05964												
Std. Dev. for sample preparation (SPREP)				0.080393												

*The design includes four conditions with 10 replicates each and three plates per replicate. The standard deviation, variance, and average are calculated (equations are provided in Supplementary Appendix 2). The standard deviations for the four conditions are pooled to yield the intermediate precision. The standard deviation for an individual plate count is calculated. The *SEM* for the average of three plate counts is calculated, which allows the determination of the standard deviation for the sample preparation (see also Table 4 and Figure 4).*

##### Uncertainties Associated With Example Lactobacillus spp.

Using the data obtained from qualification experiments (Table 3) it is possible to evaluate the measurement uncertainty for the analytical procedure, as well as uncertainty contributions from individual procedure components. For the example *Lactobacillus* spp., measurement uncertainty (*MU)* for the entire procedure, as well as uncertainty from the triplicate plating component, alone, were estimated. Equations required to calculate both uncertainties are provided in Supplementary Appendix 2.

ANOVA data yields total uncertainty for the procedure. This is known as intermediate precision (*S*_*IP*_) and consists of the pooled standard deviation from all conditions and replicates (Table 3).

The data for the triplicate plate counts informs the calculation of standard deviation for a single plate count and the standard error of mean (*SEM*) for the average of all three plate counts (Table 3). This is discussed above in section “Precision From Replicating Steps in an Analytical Procedure.”

The uncertainty contribution from triplicate plating requires the use of all procedure qualification data for all conditions and replicates. For simplicity, Table 4 shows only the values for condition 1, replicate 1. The headings in Table 4 indicate values that are required for calculating the uncertainty from plating. Supplementary Appendix 2 provide the steps to calculate triplicate plating uncertainty and the standard deviation for a single plate count. In this paper the average formula, rather than weighted, is used to determine the average plate count for one replicate. Other formulas for calculating averages in microbiology that weight the inputs according to dilutions or mass can be used when relevant. Weighted means divide the sum of all colonies counted by the sum of all volumes involved. This is a reasonable way to try to use all the information gathered to obtains the best possible density (ISO, 2020). For the example in this paper averages (unweighted means) are used because the manufacturing process is well-characterized and produces consistent product, and the analytical enumeration procedure reliably produces plates from one dilution with counts between 25 and 250. Here, weighted calculations are not needed, and the simplified approach helps to optimize workload in the laboratory. When all qualification data are included in the calculations, as required, the standard deviation for all plate counts equals 0.0962 Log_10_ CFU/g. Since the average of the three plate counts is used for ANOVA analysis, the standard error of mean ($\bar{y}$) for the triplicate plate counts is calculated by dividing by the square root of three ($\sqrt{3}$): $\bar{y}$ = 0.0962/ $\sqrt{3}$ = 0.0556 Log_10_ CFU/g (Eq. 10, Supplementary Appendix 2). This value is an estimate for the uncertainty for the triplicate plating component reflected in the reportable value.

**TABLE 4:** Determining the uncertainty of plating.

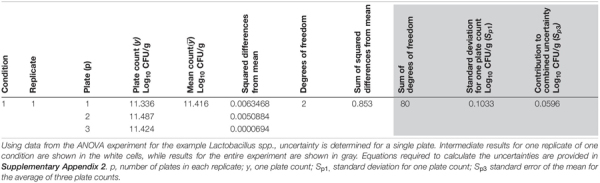

The pooled variance from the plating steps (*S*_*P3*_) can subsequently be subtracted from the pooled variance of the total procedure (*S*_*IP*_) to yield the variance for sample preparation (*S*_*PREP*_). The square root of the variance yields the estimate of the uncertainty or standard deviation for *S*_*PREP*_ (Table 3). Figure 4 illustrates the uncertainties discussed above in relation to the steps in the *Lactobacillus* spp. analytical procedure.

##### Completing the Qualification Procedure for Example Lactobacillus spp.

**FIGURE 5:**
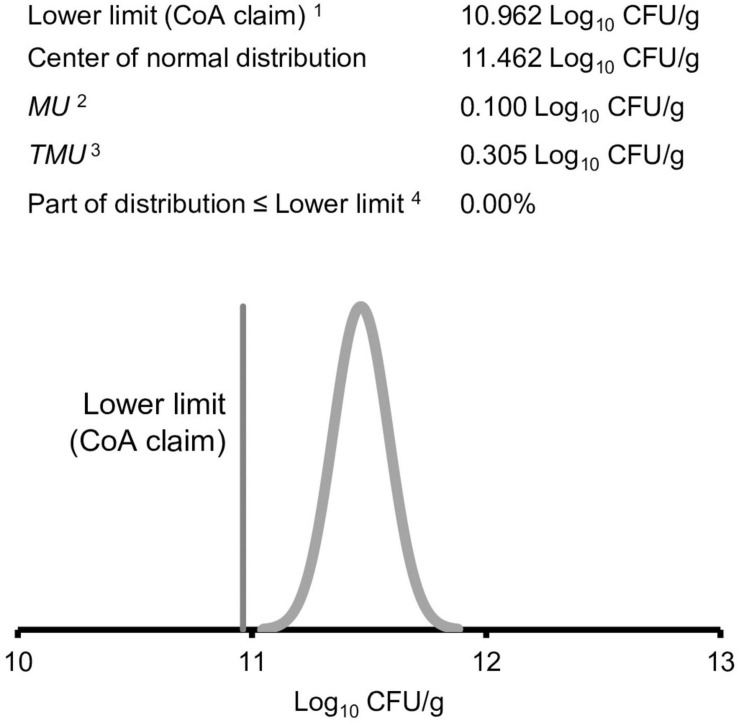
The experimental intermediate precision, *S*_*IP*,_ is used to calculate the probability of being wrong, which is <0.0%. Data from the example *Lactobacillus* spp. ^1^Certificate of analysis (CoA). ^2^Measurement uncertainty (*MU*) determined from experimental intermediate precision (Table 3). ^3^Target measurement uncertainty (*TMU*) obtained from Figure 3. ^4^Probability of being wrong <0.0% is less than the decision rule requirement of 5.0%.

It is important to assess and summarize the alignment of qualification results with the requirements of the decision rule and ATP. In qualification experiments, the uncertainty of the total procedure (intermediate precision; *S*_*IP*_) and the *MU* were determined. These qualification results, along with specifications set during development of the ATP (lower limit or CoA claim, center of normal distribution curve – lower limit plus overage, release limit, *TMU*) can be used to determine the actual probability of being wrong by adding the information to an MS EXCEL (2016) worksheet for calculating NORMDIST (Supplementary Appendix 2) or by following manual calculations in Supplementary Appendix 2. Figure 5 represents the NORMDIST calculation. Using the example *S*_*IP*_, the probability of being wrong is 0.00% This is much less than the decision rule requirement of 5%. Therefore, the company could consider increasing uncertainty (e.g., use two plates instead of three) or decreasing overage.

To complete the qualification of the analytical enumeration procedure used in the example *Lactobacillus* spp., a qualification statement must be documented. For example:

In qualification experiments, the selected analytical procedure enumerated culturable *Lactobacillus* spp. cells as specified by the ATP. Therefore, the procedure is fit for intended use.

## Procedure Comparison

Development scientists are often called upon to judge whether an existing analytical procedure is fit-for-purpose or whether an adaptation or development of a new analytical procedure is needed. The APLM process described in the proposed USP <1220> (Martin et al., 2017) provides tools to understand analytical procedures and generate and evaluate data needed to make these determinations. In the exercise of comparing two CFU analytical procedures, detailed information about enumeration procedures and data can be gathered by following the steps outlined in this manuscript. Additionally, the QRM question: “What might go wrong?” along with risk mitigations can be included in the comparison.

APLM can be used to evaluate whether both procedures in the comparison are fit for intended use. The acceptance criteria for fitness are found within the measurand, the decision rule, and the ATP.

It should be noted that available information for probiotic CFU enumeration analytical procedures that have been published or developed in a laboratory for proprietary use may be limited. In such cases, prior knowledge, expertise, literature information, or any documented information is used to select variables that should be investigated before the comparison is conducted. In all cases, a comparison should be considered carefully when full, detailed information is not available to ensure that the comparison is scientifically rational.

### Tolerance Interval (*TI*) to Compare Procedures

The performance of each enumeration procedure can be demonstrated and evaluated using *TI* as described in USP <1220> (USP, 2018). The *TI* is the given range in which a specified proportion of all future reportable values will fall. The uncertainty and average value determined during qualification for each procedure to be compared can be used to calculate the *TI*. The interval calculated for each procedure can be compared as an individual piece of information and be evaluated against the ATP. The risk analysis performed during APLM can also inform the comparison.

**FIGURE 6:**
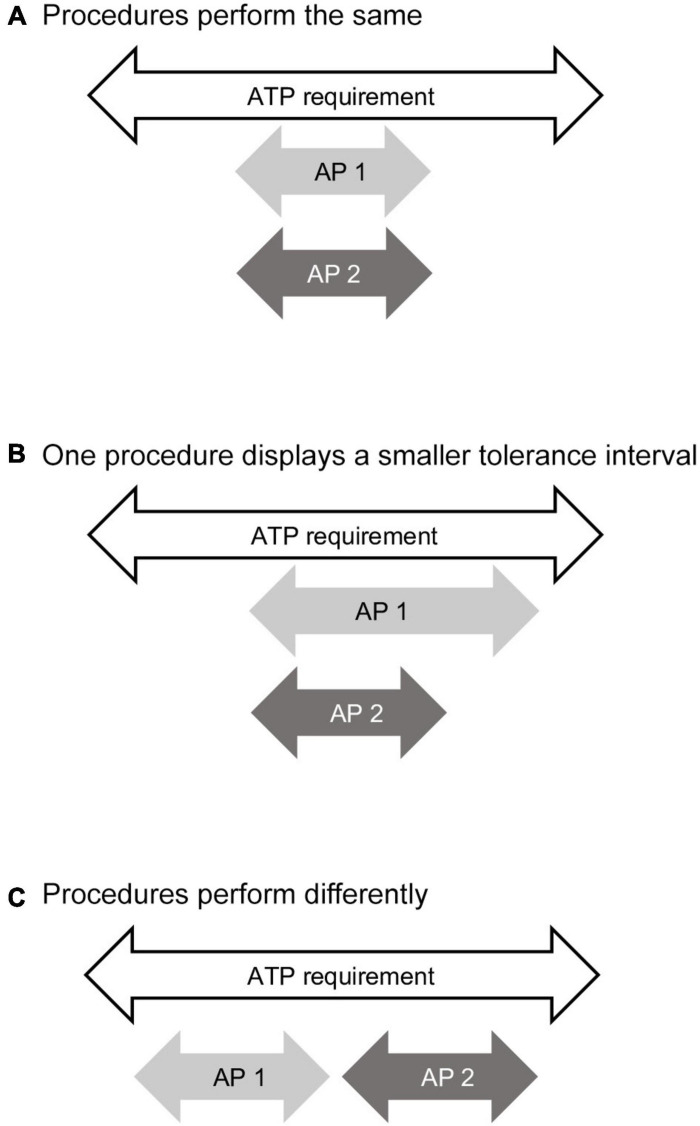
Analytical procedure comparison using the analytical target profile (ATP) and tolerance intervals (*TI*). The white arrow represents the specification range required by the ATP for the analytical procedure to be fit for use. The light gray arrow (AP 1) represents the tolerance interval for one analytical procedure. The dark gray arrow (AP 2) represents the tolerance interval for the second analytical procedure. **(A)** Comparison showing both procedures perform the same. **(B)** Comparison showing the tolerance interval for procedure 1 is larger than procedure 2. **(C)** Comparison showing the two tolerance intervals of the two procedures do not overlap. In all situations both procedures are fit for use.

Three possible outcomes from procedure comparison using the ATP and *TI* are illustrated in Figure 6. Both procedures have met the requirement of the ATP and the *TI* for both procedures are identical. It can be concluded that the procedures perform the same (Figure 6A). Both procedures fulfill the requirements of the ATP, but one procedure displays a narrower *TI* and hence a smaller measurement uncertainty compared to the other procedure (Figure 6B). Both procedures fulfill the requirements of the ATP, thus are fit for use, although the *TI* do not overlap (Figure 6C).

In the protocol to design the analytical procedure comparison, the acceptability of one or all three outcomes must be stated. The acceptability of outcome A, in which the procedures perform the same, is straightforward. Future values from both procedures can be compared directly. For outcome B in which one procedure is different from the other, but the *TI* overlap, the end-user must decide whether this difference in performance is acceptable. In outcome C, the analytical procedures perform differently. There is no overlap in the *TI*. The difference in future results needs to be accounted for.

#### Bias and Tolerance Interval (*TI*)

The *TI* considers both accuracy (bias) and precision. For microbiological CFU procedures, bias may not be able to be determined, as discussed above. Therefore, the *TI* may not overlap, simply due to distributional uncertainty of the test samples used during qualification experiments. The protocol for comparing the methods must take this into account.

#### Tolerance Interval (*TI*) Comparison of Two Procedures for the Example *Lactobacillus* spp.

In this comparison, procedure A will represent information and data simulated for the example *Lactobacillus* spp. The second procedure, procedure B, uses a different plating agar and a different mixing technique for the initial sample suspension. To demonstrate the use of *TI*, values for procedure B were simulated.

First, the measurand, decision rule, and ATP for procedures A and B are compared to ensure the procedures are designed for the same purpose. Second, the results of the risk analyses are reviewed to confirm the procedures can be compared. If the outcome of these evaluations stipulates the two procedures are comparable, an actual comparison of the procedures performances is made based on data gathered during qualification experiments. If this data is not available, information used to inform the decision of fit for intended use must be closely scrutinized. It is possible, in some cases, to apply historical data to gather information and make calculations needed to develop ATP requirements. However, before proceeding, it may be necessary to design and conduct qualification experiments, such as the ANOVA experiments described in this paper.

Using data that is acceptable to informing the ATP, the *TI* for each procedure is calculated and compared. The data for procedure A was taken from condition one in the *Lactobacillus* spp. example (Table 3). Data for procedure B was simulated to represent one condition from qualification experiments. The *TI* calculations needed for comparison are shown in Table 5. It should be noted that the factor *K* used in *TI* calculations is not the *k*-factor used in uncertainty calculations. Both equations needed to calculate *TI* and *K* are found in Supplementary Appendix 2.

**TABLE 5:** Comparing procedure performance by tolerance intervals (*TI*).

*TI* calculations for procedure A (Log_10_ CFU/g)				
**Condition**	$\bar{C}$	S_*C*_	***TI***	
			$\bar{C}-\left(K\times S_{C}\right)$	$\bar{C}\left(K+S_{C}\right)$

A-1	11.491	0.108	11.218	11.765

***TI* calculations for procedure B (Log_10_ CFU/g)**				

B-1	11.442	0.126	11.123	11.761

*TI calculated for one ANOVA condition of procedure A (A-1) and B (B-1), respectively. $TI=\bar{C}\pm \left(K\times S_{C}\right)$, where $\bar{C}$ = mean value for the condition. *K* = coverage factor (90% confidence, 90% coverage, *n* = 10) = 2.535. *S*_*c*_ = Standard deviation for each condition. K values for *TI* calculations can be calculated as described in Supplementary Appendix 2. $\bar{C}$, average for one condition; *S*_*C*_, standard deviation for one condition; *K*, tolerance interval factor.*

For this comparison, both procedures are deemed fit for intended use; both meet ATP requirements. The *TI* show a substantial overlap. Therefore, both procedures could be used; the end-user needs to decide whether the difference in performance is acceptable.

## Real-Life Data for *Lactobacillus acidophilus* Powder

To illustrate the practical use of the APLM for CFU procedures, the tool was applied to the analysis of real-life data from a probiotic manufacturer’s development laboratory. The laboratory was investigating the enumeration of a probiotic *Lactobacillus acidophilus* powder. The ANOVA design includes five conditions with 10 replicates each. Each replicate consists of three plates generated as per proprietary procedure. The conditions capture modifications to the procedure. Condition details and the ANOVA analysis table of are included in Supplementary Appendix 4.

Although study parameters used in the manufacturer’s development laboratory and the simulation are different (analytical procedures; ANOVA designs—number of conditions, details varied), it is interesting that the standard deviations, variances, and standard errors determined via the ANOVA analysis are smaller for the real-life data than those of the simulation. This observation may align with conservative estimates knowingly used when designing the simulation. The data shows that variations occur when changes are made to the procedure. This underscores the need for qualification and comparison tools such as APLM and *TI*. For complete application and maximized impact to the probiotic industry, utilization of the complete APLM approach, including the ATP, measurand definition, risk analysis, and comparison with *TI* described in USP <1210> (USP, 2018) is recommended.

## Discussion

Through information acquisition, educated decision-making, and documenting key requirements, APLM creates a fluid knowledge base that becomes the cornerstone of communication for all discussions regarding an analytical procedure and the product(s) it supports. This type of qualification (validation/verification) management system ensures an analytical procedure is and remains fit for intended use throughout its lifecycle. Moreover, the APLM process enhances organizational communications regarding the status quo or changes to the procedure that will improve the quality assessment of the probiotic products it supports. Process transparency, efficient knowledge transfer, and routine use of appropriate analytical procedures makes authenticating label claims and meeting stakeholder expectations easier for all parties involved.

Both the qualification of procedures under APLM and comparison by *TI* hold the potential to add flexibility in determining the quality and release of probiotic products. The insights gained through *TI* comparison, taken along with APLM information, can inform decisions regarding the procedure itself (Is it performing as desired? Should it be modified or replaced?). Details captured while conducting the procedure, such as the effects produced by selecting the product of one supplier over another, analysis errors caught midstream, or replacement of equipment used routinely for the procedure can also be observed. Changes made in the manufacturing process may or may not be reflected in the product quality and hence the reportable values of the analytical procedure used to test the end-product. Either way, changes are documented in the system and new results can be compared to prior results to evaluate the impact to product quality. The information compiled can point to equivalencies and differences; again, providing flexibility and improving product quality.

Other strategies, tools, and guidelines are available to meet GMP regulatory requirements surrounding the use of scientifically valid procedures. For example, the probiotics industry is aware of and uses quality by design, risk analysis, control strategies, and validations guided by the United States Food and Drug Administration (FDA), Health Canada, the World Health Organization (WHO), and others. These programs are predecessors or foundation blocks that have been applied to lifecycle management. When APLM is applied to CFU enumeration, elements of predecessor programs can be used to help detail steps within the approach, but the path forward and elements required to complete each step are streamlined. For companies that have already selected alternative options to meet regulatory requirements and fit their needs, APLM can be used as a complementary approach. Depending on experience with design and/or qualification of procedures, the combination of information gained through predecessor programs and APLM/*TI* can be used to build flexibility. If a company chooses to switch to APLM, much of their historical data would support the steps and requirements within the approach. For new companies, APLM and this manuscript provide a step-by-step procedure to meet regulatory requirements. APLM uses tools and practices of good science and metrological principles. The APLM process is streamlined, logical, and organized. It is focused on ensuring the reportable value is fit for intended and uses many documented and accepted practices expressed in recognized standards. Moreover, the lifecycle view of analytical development facilitates the analytical procedure to follow the development status of the product, which is especially important during the transition from development to final product.

High quality products require high quality analytical procedures and performance control to ensure efficient evaluation and monitoring of product quality. As outlined in this article, using the ATP, APLM, and *TI* comparison will allow probiotic manufacturers to define, control, monitor, and compare procedure performance. This is illustrated by revisiting the USP (2020) definition: “The ATP is a prospective description of the desired performance of an analytical procedure that is used to measure a quality attribute, and it defines the required quality of the reportable value produced by the procedure.”

## Conclusion

APLM ensures that an analytical procedure is fit for its intended use. Pertinent information must be gathered, understood, and experimentally explored before procedures are applied for product quality assessment. This includes a thorough risk analysis and complete control strategy. The APLM approach and understanding it provides can also be used to effectively document and compare analytical procedures using well-evaluated measurement uncertainty and statistical tools such as tolerance intervals. This type of comparison has a broad range of applications.

The comparison exercise using APLM information and *TI* calculations showed that *TI* can be used to identify agreements and anomalies. The *TI* set expectations for the usefulness of analytical procedures. If the procedure(s) are deemed fit as per the APLM ATP, then one needs only to compare TI limits or endpoints, when considering continued fitness, need for modification, or whether a change in procedure(s) may be beneficial. These comparisons can be conducted over the life cycle of the procedure.

Better understanding and control of analytical procedures will improve the quality of results that the probiotics industry uses to control and improve processes that lead to the delivery of quality products to consumers. It will also improve the quality of results used for making business decisions and supporting claims of dose and, therefore, health benefits. Accumulated, detailed knowledge gathered through APLM and *TI* comparisons will drive innovation in the probiotics industry.

## Data Availability Statement

The raw data supporting the conclusions of this article will be made available by the authors, without undue reservation, to any qualified researcher.

## Author Contributions

BK, JS, MW, and VG wrote and edited drafts and revisions of the manuscript. CP, CV, MP, and PB edited the revisions. JS, MW, and MP drafted the initial risk analysis. MW contributed to all statistical analyses and developed the EXCEL workbook for the project. CV refined, formatted, and compiled all figures and tables. MP contributed to the data examples to enhance discussion of project topics and improve example parameters. JS compiled author contributions and edited to form the final manuscript. All authors contributed to the discussions of project topics, edited the abstract, and read and approved the final manuscript.

## Conflict of Interest

All authors are members of the USP Probiotics Expert Panel. Some authors are employed by companies in the biotherapeutics or probiotics industry: CV and CP are employed by Bacthera and MP is employed by Probiotical Research srl. MW (Consultant), PB (Advanced Microbial Analytics Research Solutions) and JS (Eurofins Microbiology Laboratory) provide services or work for companies that provide services to the biotherapeutics or probiotics industry. VG and BK are employed by the United States Pharmacopeial Convention, which operates in the absence of any commercial or financial relationships that could be construed as a potential conflict of interest.
