# Supplementary material for: Improving and Comparing Probiotic Plate Count Methods by Analytical Procedure Lifecycle Management
Source: Front Microbiol. 2021 Jul 12;12:693066. doi: 10.3389/fmicb.2021.693066 (PMC8312684; doi:10.3389/fmicb.2021.693066)
Supplement: Supplementary file 1 [file Data_Sheet_1.pdf]

Appendix 1: Terms and definitions

Term	Symbol	Definition / Explanation
Analytical procedure lifecycle management	APLM	A management system for analytical procedures that incorporates all events that take place over the procedure life cycle that are designed to demonstrate that a procedure is, and remains, fit for the intended purpose.
Analytical target profile	ATP	Objectives and requirements for performance of the analytical procedure and stipulates the quality of reportable value(s).
Coverage factor	k	Number larger than one by which a combined standard measurement uncertainty is multiplied to obtain an expanded measurement uncertainty.
Critical difference		The 95% confidence interval limit for the absolute difference between two test results obtained under the reproducible conditions.
Decision rule		Defines the use of the reportable value.
Decision unit		The material from which a sample is collected and to which an inference is made.
Distributional uncertainty		Uncertainty resulting from intrinsic variability associated with the distribution of microorganisms in the sample, the initial suspension, and subsequent dilutions.
Expanded uncertainty	U	Quantity defining an interval about the result of a measurement that may be expected to encompass a large fraction of the distribution of values that could reasonably be attributed to the measurand.
Guard band		The magnitude of the offset from the specification limit to the acceptance or rejection zone boundary.
Intermediate Precision		Closeness of agreement between test results obtained with the same method on the same or similar test materials in the same laboratory with different operators using different

		equipment on the same or similar test materials in the same laboratory with different operators using different equipment. Intra-laboratory reproducibility is the same as intermediate precision.
K factor used for tolerance interval	K	Factor used to calculate the tolerance interval.
Matrix uncertainty		Uncertainty resulting from a test portion that is not truly representative of the laboratory sample.
Measurand		The quantity subjected to measurement.
Measurement uncertainty	MU	Non-negative parameter characterizing the dispersion of the quantity values being attributed to a measurand, based on the information used. The parameter may be, for example, a standard deviation called standard measurement uncertainty.
Reproducibility		Reproducibility can have different meanings in different references.<br><br>It can mean the variation between laboratories (inter-laboratory reproducibility).<br><br>It can mean the variation of results within a laboratory (intra-reproducibility). This is the same as intermediate precision.<br><br>This paper uses the term intermediate precision and does not discuss inter-laboratory reproducibility.
Standard error of the mean	SEM	A measure of the dispersion of sample means around the population mean. The *SEM* takes the standard deviation (SD) and divides it by the square root of the sample size.
Standard uncertainty	u	Standard uncertainty of measurement expressed as a standard deviation.
Target measurement uncertainty	TMU	Measurement uncertainty specified as an upper limit and decided based on the intended use of measurement results.

		The greatest allowable value for the standard deviation of the reportable value, generated by the analytical procedure, that still allows the procedure to be considered fit for intended use.
Technical uncertainty		Uncertainty resulting from operational variability associated with the technical steps of the analytical procedure.
Tolerance Interval	*TI*	A statistical interval within which, with some confidence level, a specified proportion of a sampled population falls. The endpoints of a tolerance interval are called tolerance limits. An application of tolerance intervals to manufacturing involves comparing specification limits prescribed by the client with tolerance limits that cover a specified proportion of the population.
Variance		The square of the standard deviation.
