# Supplementary material for: Improving and Comparing Probiotic Plate Count Methods by Analytical Procedure Lifecycle Management
Source: Front Microbiol. 2021 Jul 12;12:693066. doi: 10.3389/fmicb.2021.693066 (PMC8312684; doi:10.3389/fmicb.2021.693066)
Supplement: Supplementary file 2 [file Data_Sheet_2.pdf]

Appendix 2: Equations and MS EXCEL Formulas

Equation Symbols:

For the purposes of this paper, the following symbols apply.

$\bar{C}$	average for one condition
h	the index of the condition $(h = 1, 2, ..., N)$
i	the index of the replicate $(i = 1,2, ..., r)$
j	the index of the plate $(j = 1, 2, ..., p)$
k	coverage factor
K	tolerance interval factor
n	number of values used to calculate $\bar{Y}$
N	number of conditions
p	number of plates in each replicate
P	desired probability value
pr	acceptable probability of making a wrong decision
r	number of replicates in each condition
S_C	standard deviation for one condition
S_C^2	variance for one condition
S_{IP}	pooled standard deviation for all conditions (intermediate precision)
S_{p1}	standard deviation for one plate count

S_{p3}	standard error of the mean for average of three plate counts
S_{PREP}	standard deviation for sample preparation
TI	tolerance interval
TMU	target measurement uncertainty
U	expanded uncertainty
$\chi^2_{\propto:n-1}$	a chi-squared percentile with area α to the left and $(n\text{-}1)$ degrees of freedom can be found in tables available online (https://www.itl.nist.gov/div898/handbook/)
y	one plate count
$\bar{y}$	average of plate counts for one replicate
$\bar{Y}$	average value used to calculate the tolerance interval
$Z^2_{(1+P)/2}$	the square of the standard normal percentile with area (1 + P)/2 to the left

TMU EQUATION

Equation 1 $$TMU = U/k_{pr}$$ (Burgess et al., 2016)

MS EXCEL Formula for Determining the Probability of Being Wrong

The probability that a reportable value will be less than the TMU can be calculated using the NORM.DIST function in EXCEL. The TMU in the function is varied until the desired probability is achieved. This probability is the acceptable probability of being wrong from the decision rule. For TMU with a lower limit, only, the formula is:

Equation 2 =NORM.DIST(lower limit, X, TMU, TRUE)

The lower limit is the value of the specification at the low limit.
X is the mean, the center, or mid-point of the distribution.

The value of the TMU is varied until the formula returns the desired probability below the lower limit.
TRUE returns the cumulative distribution function.

ANOVA EQUATIONS

There are N conditions. For each condition, there are r replicates with p_j ($j=1, 2, ..., p$) plate counts for replicate i, resulting in y_j $\log_{10}$ CFU/g for plate count j of replicate i.

In the *Lactobacillus* spp. example, there are four conditions (N=4) with 10 replicates per condition (r=10 or r_i where each replicate is indicated by i=1,2, 3…, or 10). Each replicate is plated in triplicate (p=3) and each plate is identified as j=1, 2, or 3.

Average of plate counts for one replicate

To calculate the average of plate counts for one replicate associated with the *Lactobacillus* spp. example in this paper, the following caveats apply:

1. Data includes at least about 20 sets of replicates (e.g., 20 sets of triplicate plate counts).
2. The data covers a small range (small is < an order of magnitude).
3. The data variability is similar (small is < order of magnitude).

Equation 3

$$\bar{y} = \frac{\sum_{j=1}^{p} y_j}{p}$$

Average for one condition

Equation 4

$$\bar{C}_h = \frac{\sum_{i=1}^{r} \bar{y}_i}{r}$$

Standard deviation for one condition

Equation 5

$$S_C = \sqrt{\frac{\sum_{i=1}^{r} (\bar{y}_i - C_h)^2}{r - 1}}$$

Variance for one condition

Equation 6 $$S_C^2$$

Pooled standard deviation for all conditions (Intermediate precision)

Equation 7 $$S_{IP} = \sqrt{\frac{\sum_{h=1}^{N}(n_h - 1) \times S_C^2}{\sum_{h=1}^{N}(n_h - 1)}}$$

Average plate count for one replicate

Equation 8 $$\bar{y}_i = \frac{\sum_{j=1}^{p_i} y_{ij}}{p_i}$$

Standard deviation for a one plate count based on all replicates in all conditions (*p = 40*)

Equation 9 $$S_{p1} = \sqrt{\frac{\sum_{h=1}^{N}\sum_{i=1}^{r}\left(y_{ij} - \bar{y}_i\right)^2}{p}}$$

Standard error of the mean for the average of three plate counts (*p* = 3)

Equation 10 $$s_{p3} = \frac{s_{p1}}{\sqrt{p}}$$

Standard deviation for sample preparation

Equation 11 $$S_{PREP} = \sqrt{S_{IP}^2 - S_{p3}^2}$$

$\boldsymbol{TI}$ EQUATIONS

Equation 12 $$TI = \bar{\boldsymbol{C}} \pm (K \times S_c)$$

Equation 13 $$K = \sqrt{\frac{Z^2_{(1+P)/2} \times (r-1)}{\chi^2_{\alpha:r-1}} \times \left(1 + \frac{1}{r}\right)}$$
