# Supplementary material for: Improving and Comparing Probiotic Plate Count Methods by Analytical Procedure Lifecycle Management
Source: Front Microbiol. 2021 Jul 12;12:693066. doi: 10.3389/fmicb.2021.693066 (PMC8312684; doi:10.3389/fmicb.2021.693066)
Supplement: Supplementary file 3 [file Data_Sheet_3.pdf]

Appendix 3: Risk analysis for CFU analytical procedures and for the Example *Lactobacillus* spp.

This appendix is intended to be a guide for identifying potential risks, their potential impacts on the reportable value and performance, and control strategies that apply to analytical CFU procedures. The risks are listed and grouped according to the ISO categories (technical, matrix, and distributional), which are defined in standard 19036:2019 (ISO, 2019). Due to limited number of CFU or proliferation-based reference standards with assigned, certified, or reference values available, measurement and evaluation of the accuracy (or bias) of a CFU procedure has not been included. However, variables in the procedure may still affect the accuracy of the measurement. Possible mitigation strategies are given to further describe the risks and to address how they can be controlled.

The table includes risk information that is applied to CFU procedures, in general, and specifically to the *Lactobacillus* spp. example. The table applies to a bulk product. Although many of the risks in the table would be applicable to a finished product and/or other matrices, additional risk components may be recognized and included. The analytical procedure for the example, and therefore the risks included in the table, was based on a fully developed USP monograph that included instructions based on the submitting company's practices. The right-hand columns of the table contain risks and controls that apply specifically to the *Lactobacillus* spp. example. There is also a column indicating whether the risk was included in the example ANOVA study. Although the risks associated with analytical CFU procedures were examined in detail, this table is not comprehensive. The user may encounter risks that have not been included in the table or may find that not all listed risks are applicable to the analytical procedure being investigated. The reader is encouraged to use this table as a guide when identifying potential risks for a specific CFU enumeration procedure.

Risk or Uncertainty Component	Impact	Details of the Risk	Mitigation Strategy	Assessment for *Lactobacillus* spp. Example	Include in *Lactobacillus* spp. ANOVA study?
**ISO Risk Category[a]: Technical**					
Analyst	Accuracy<br><br>Precision	Variability in carrying out the procedure	Competent analysts<br><br>with education and training to meet GMP	Company analysts meet GMP expectations	Yes<br><br>Different analysts included
Day-to-day variation	Accuracy<br><br>Precision	Between day variability can be significant due to climate changes, equipment performance, different reagent lots, and sample stability	Meets GMP	Meets GMP	Yes<br><br>Procedure conducted on different days
Procedure	Accuracy<br><br>Precision	If options or variations are allowed in the procedure, there are work instructions that describes when specific variations can be used and how they are applied	Written instructions are clear and supported by data	The laboratory procedure has been clearly written to provide the instructions required to follow company practices and steps that may vary	No<br><br>The company procedure is self-explanatory and will be followed for each experiment

Use of the analytical procedure over a long period of time	Accuracy<br><br>Precision	Laboratory conditions can vary over a year and impact procedure performance	Monitor results with trend analysis and use control charts whenever possible<br><br>Use of trends and charts is part of continued verification (Stage 3 in the Lifecycle)	Use control chart for triplicate plate results<br><br>Track reportable values	No<br><br>ANOVA experiments are conducted over short time periods
Plating practices/Selection of plating technique	Accuracy<br><br>Precision	Variation in growth can be observed between pour (single and/or double layer) and surface plating depending on the oxygen tolerance of the species being cultivated<br><br>Use of membrane filters can lower counts<br><br>Agar quality (composition, wetness, lumps, etc.) affects counts	Use of scientific and expertise knowledge, experience, and specific instructions<br><br>Implement a quality control program for all media used in enumeration procedures	The plating technique, including acceptable quality of agar to be used, is described in the company procedure<br><br>Control programs are in place	No
Selection of suspension/rehydration, diluting, and plating media	Accuracy<br><br>Precision	Composition of the media should maintain viability and/or support ability to culture microorganisms in the product	Use of scientific knowledge, expertise, experience, and specific instructions	The media are specified in the procedure and have been validated for use with the powder	No

Ingredient from unqualified or alternative qualified source used in suspension/rehydration, diluting, or plating media	Accuracy<br><br>Precision	Impact on total CFU counts	Ingredient sources are qualified by R&D during procedure development	The source of ingredients is specified in the company procedure	Yes<br><br>Different sources of ingredient will be included in lots used for ANOVA studies
Incorrect ingredient for various media listed in SOP/work instruction for culture medium	Accuracy<br><br>Precision	Impact on total CFU counts	Multiple people check the documentation of procedures during reviews leading to approval	The ingredients listed in the company procedure are correct	No
Ingredient measured incorrectly when making media	Accuracy<br><br>Precision	Impact on total CFU counts	Analysts are trained in media production to meet GMP	The company’s analysts have been trained to make media	No
Shelf life of suspension, dilution, and plating media	Accuracy<br><br>Precision	Storage conditions (e.g., time and temperature) for all media used can affect the overall ability of the procedure to support CFU formation	Control shelf-life<br><br>Storage conditions can be defined based on shelf-life studies	Shelf life of all media involved in the procedure have been defined	No
pH all media used in the procedure	Accuracy<br><br>Precision	Media pH can influence the overall ability of the procedure to support CFU formation	Determine optimum pH during procedure development and adjust media	pH is defined for media used in the procedure	Yes<br><br>The company has two pH meters, which will be used during media

		Optimum pH for media can be strain dependent	production lots accordingly<br><br>Use a multi-point calibrated pH meters when adjusting<br><br>Ensure instrument calibration and analyst training meets GMP expectations	pH meters are calibrated<br><br>The calibration technique clearly described in and SOP	production for the study
Autoclave	Accuracy<br><br>Precision	Contamination not inactivated<br><br>Media components destroyed or inactivated	To meet GMP, specify autoclave load, configuration for input (e.g., flasks, test tubes), define times and temperatures, etc.	Conditions for autoclaving are defined for the media in this study	Yes<br><br>Different autoclaves are used
Balance	Accuracy<br><br>Precision	Balance not calibrated<br><br>Incorrect amount taken	Detailed instructions for balance verification and analyst training meet GMP	Meets GMP	Yes<br><br>Different balances are used
Pipettes – volumetric or serological	Accuracy<br><br>Precision	Incorrect volumes used in media preparation, sample preparation, suspension/rehydration, dilution, and plating	Meet GMP by supporting the maintenance and calibration of pipettes per	The company uses disposable serological pipettes that are confirmed accurate by	Yes<br><br>Different lots of serological pipettes are used

			specified schedule(s)<br><br>Use certificates of warranty to confirm accuracy for disposables<br><br>Include pipetting in analyst training and competency checks	certificate of warranty<br><br>Analysts are properly trained in pipetting<br><br>The company meets GMP	in sample preparation
Pipettors with tips	Accuracy<br><br>Precision	Incorrect volumes used in media preparation, sample preparation, suspension/rehydration, dilution, and plating	Calibrate pipettors with tips before use to meet GMP<br><br>Included pipetting in analyst training and competency checks	The company meets GMP for pipettor calibration and analyst training	Yes<br><br>Different pipettors and tips are used during dilution and plating
Graduated cylinders	Accuracy<br><br>Precision	Incorrect volumes used in media, sample preparation, and/or suspension/rehydration	Ensure the cylinders are not damaged<br><br>Schedule accuracy checks for cylinders that are used and/or sterilized frequently<br><br>Train analysts to correctly read graduated cylinders	The company meets GMP for use of graduated cylinders	No<br><br>The same set of graduated cylinders is used throughout the experiments

Other equipment	Accuracy<br><br>Precision	Depends upon laboratory procedures	Depends upon laboratory procedures	No “other equipment” was required in the company procedure	No
Instability of sample	Accuracy<br><br>Precision	Over time and regardless of handling, storage, etc., the nature of the sample changes because microbial cells change throughout their lifecycle	Clear, detailed instruction from gathering sample (include during which part of life the test is made and/or consider when developing the measurand) to reporting result are documented to help control stability<br><br>Meet GMP	The company meets GMP as demonstrated by passing GMP audits	No
Storage of sample	Accuracy<br><br>Precision	Must provide conditions to keep sample as stable as possible before and perhaps during/after use	Control temperature and relative humidity<br><br>Specify acceptable temperature and relative humidity ranges<br><br>Meet GMP	The company meets GMP and is attentive to the conditions that samples are exposed to	No

Preparation of test portions or test solutions	Accuracy<br><br>Precision	Impact on total CFU counts	Provide detailed instructions on preparation (e.g., portion weights and volumes must be adequate)<br><br>Specify and control temperature and time for suspension and dilution	Clear, detailed instructions are in included in the analytical procedure	No
Homogenization of sample	Precision	Must release cells from matrix but not kill them	Specify technique and/or equipment to be used such as mechanical blender, stomacher, vortex, hand maceration	Specified in company's analytical procedure	No<br><br>Only one blender was used to homogenize all samples
Increased or decreased sample size	Accuracy<br><br>Precision	Sample size affects standard error<br><br>Colony count will change and affect variance of mean count	Sample size to be standardized during procedure development<br><br>Analyst training includes weighing and measuring	Sample size is stated in procedure and analysts are trained to meet GMP	No
Hold times	Accuracy<br><br>Precision	Suspension/rehydration, dilution, time until agar is added, incubation time, etc.	Specify all hold times	The company determined all hold time during procedure development	No

		Determine whether plates can be held in refrigerator before counting and, if so, how long			
Dilution	Precision	Uncertainty may depend upon dilution scheme	Specify dilution scheme and/or instruct to record dilution scheme	Dilution scheme is specified	No
Serial dilution	Accuracy<br><br>Precision	Aseptic technique is required to prevent cross contamination	Sterile consumables and a protected work area<br><br>Analysts trained in aseptic technique	The company provides sterile consumables and a protected work environment<br><br>Analysts are trained in aseptic technique meet GMP	No
Heat shock	Accuracy<br><br>Precision	Some spore forming bacteria need a heat shock to form colonies on counting medium	Instructions for heat shock are detailed and clear<br><br>Instructions for heat shock include specific hold times and temperature(s)<br><br>Water bath meets GMP	Not relevant for *Lactobacillus* spp. example	No

Pipetting technique	Precision<br><br>Accuracy	Many pipetting variables will affect uncertainty, e.g., mixing between transfers, vortex speed and time of mixing, use of fresh tips, washing the pipet or tip when dispensing	Training on proper pipetting<br><br>Specify pipettes or pipettors and tips<br><br>Standardize practices<br><br>Ensure calibrated pipettes and pipettors with tips are used<br><br>Meet GMP	The company has a pipetting training module for analysts pipetting and has detailed the techniques required for this procedure<br><br>Meets GMP	Yes<br><br>This is captured by using different analysts and various pipettes and pipettors with tips
Replications/plating designs	Precision	Single plate or multiple plate design - replication can impact precision	Clearly describe in which steps replication occurs, e.g., specify the number of plates generated per dilution	The company procedures state the use of a triplicate plating design	No<br><br>The same plating design is used throughout
Incubators and incubation temperatures	Precision	Incorrect incubation temperature impacts growth<br><br>The effect of temperature on CFU production may be strain independent<br><br>At “maximum load”, incubator temperature	Incubators meet GMP requirements<br><br>Temperature ranges are specified for individual incubators	Temperature ranges have been specified for all incubators	Yes<br><br>Selected incubation temperatures ranges are not varied, but different incubators are used

		must remain within its specified range<br><br>Heating and ventilation cycles are used to generate and maintain temperature ranges			
Incubation time	Accuracy	Time of incubation impacts the counts and may be strain dependent	Provide the specific time or time range of incubation	Incubation time ranges are specified	No<br><br>Plates are removed from the incubator within the specified range
Atmospheric modifiers	Accuracy<br><br>Precision	Atmospheric conditions (aerobic, anaerobic, microaerophilic) affect growth<br><br>The use of different gas generating systems, including sachets, and indicators, can affect growth	Specify whether a modified atmosphere is required for optimum growth and describe how to produce the condition(s)	Atmospheric conditions are included in the details of the procedure	No<br><br>Conditions specified in the company procedure are followed
Volumes used	Precision	Volumes effect the reportable value - transfer, dilution blank, test portion, poured agar, etc.	Specific instructions<br><br>Training programs<br><br>Meet GMP	Volumes and how to measure them are specified in the procedure<br><br>Analyst training and quality control	No<br><br>Volumes were not varied

		The range of agar volume poured needs to be adequate for growth<br><br>Hand-poured volumes can be estimated by height (a skill acquired through practice)<br><br>Repeatability of filling and emptying measuring device<br><br>Specifications of glassware and temperature effects on glassware		programs meet GMP	
Counting error	Precision	Counting of plates is critical - both manual and automatic counting are subject to error	Ensure appropriate procedure development and qualification<br><br>GMP training to include what/what not to count, range of counts, how to report counts (rounding, significant figures, Log transformation, digital and/or manual requirements), whether to adjust counts based on	The company provides extensive training for counting	Yes<br><br>Although counting error was not specifically added in the data, the impact of the variable is captured by using different analysts

			confirmation, how to determine if all counts within counting range are equally good, what to do if CFUs do not look like 10x dilutions on the plates, etc.<br><br>Qualification of counting instruments and/or tools<br><br>Address personal uncertainty – adjust group or laboratory uncertainty based on an “expert counter within the group”; develop a second count scheme to be followed by control chart; consider using mean value as a reference		
Environmental contamination	Accuracy<br><br>Precision	Possible air contamination via laboratory/building ventilation, dock doors, lack of sample	Meet environmental requirements of GMP	The company meets GMP	No

		containment during preparation, etc.<br><br>Inadequate cleaning and sanitation regimes for surfaces			
Calculation	Accuracy<br><br>Precision	Decide whether the exact product weight in a step should be used for the calculation<br><br>Ensure calculations are correct	Determine if exact volume impacts reportable value<br><br>GMP/Training – counting (see above examples) and documentation; data review (include check of all steps in manual calculations; validate automatic counts as described in standard operating procedures)	Included in analytical procedure	No
Microbial metabolism	Accuracy<br><br>Precision	Metabolic by-products that can cause competition between colonies – production of acid, antibiotics, other<br><br>Note: These impacts or risks are often unpredictable and	Functioning quality assurance program<br><br>Meets GMP<br><br>Adequate level of expertise at bench	Meets GMP	No

		cannot generally be corrected mathematically	Uncertainty does not include spurious errors and blunders		
**ISO Risk Category: Distributional**					
Mixing steps	Precision<br><br>Accuracy	Any mixing must be adequate<br><br>For example, low counts may occur if agar swirling technique does not distribute cells evenly or result in spilling agar/sample over the edge of the plate or into the lid<br><br>For example, variable counts may occur if test tubes are not uniformly vortexed to distribute cells	Provide descriptive written and verbal instructions<br><br>Demonstrate and train techniques and use of equipment<br><br>Use video recordings to illustrate techniques	The company uses video enhanced training modules	No
Distribution of microbes	Precision	Lack of homogeneous distribution results in variable counts whether uneven distribution occurs in the decision unit (entire batch or lot), a laboratory sample (subsample of the decision unit) or the test portion	Distribution problems within the decision unit may be limited in powders but increase in finished products<br><br>Homogeneity can be experimentally validated, but	The company conducted studies to show the laboratory sample is adequately homogenous<br><br>The company has written an SOP describing how to mix various	No

			validation may not hold from batch to batch<br><br>Sometimes increments are taken from like-product decision units and blended at the manufacturing level to create a primary sample<br><br>Mixing/blending is sometimes used to improve homogeneity	laboratory samples (including powders) and produce test portions	
**ISO Risk Category: Matrix**					
Laboratory sample matrix (inhibition or promotion or neutral)	Accuracy<br><br>Precision	The matrix is defined in the measurand and ATP<br><br>The matrix can impact the medium pH, component solubility, viscosity, etc.<br><br>The matrix can affect microbial cells within sample during storage and impact the analysis results	Control the procedure<br><br>Any changes to the procedure can affect the matrix and vice versa	The matrix is defined	No<br><br>One laboratory sample is used for all test portions in the experiments

Interaction of matrix and probiotic	Accuracy<br><br>Precision	Matrix may absorb, contain high concentrations of particles and/or solids which lower recovery<br><br>The matrix may be inhibitory	Matrix and maceration experimentation will define the extent of this risk	Studies have shown this is not an issue	No
Condition of microorganisms (viable and vital, sub-lethally damaged, non-culturable)	Accuracy<br><br>Precision	The matrix can impact the total counts of microorganisms		Matrix has been shown to not impact *Lactobacillus spp.*	No
Effects of competitive organisms on the recovery of specific types	Accuracy<br><br>Precision	The matrix can impact the total counts of desired microorganism		Matrix does not include other organisms	No
**Other Uncertainty Components**					
		The laboratory should consider if there are any unique steps in their procedure or unique properties of the decision unit matrix that could impact the result		No unique steps	No

[a]As defined in ISO 19036:2019 (ISO, 2019)
