# Supplementary material for: Improving and Comparing Probiotic Plate Count Methods by Analytical Procedure Lifecycle Management
Source: Front Microbiol. 2021 Jul 12;12:693066. doi: 10.3389/fmicb.2021.693066 (PMC8312684; doi:10.3389/fmicb.2021.693066)
Supplement: Supplementary file 4 [file Data_Sheet_4.pdf]

Appendix 4: The ANOVA experimental design and data for procedure qualification using real-life data for *Lactobacillus acidophilus*.

This appendix presents the ANOVA table for a real-life example. The real-life analytical procedure is an in-house method for enumerating characteristic microorganisms. The ANOVA design includes five conditions with 10 replicates each. Each replicate consists of three plates. The conditions capture procedure modifications that were investigated.

**Procedure Modification**	**Condition 1**	**Condition 2**	**Condition 3**	**Condition 4**	**Condition 5**
Enumeration medium	MRS[a]	LTP + G[b]	MRS	MRS	MRS
Hold after 1st suspension of sample	None	None	30 min	None	None
Dilution solution temperature	Room Temperature	Room Temperature	Room Temperature	5°C	Room Temperature
Pipetting tool	1 ml Pipettes and Bulb	1 ml Pipettes and Bulb	1 ml Pipettes and Bulb	1 ml Pipettes and Bulb	P1000 ART Aspirator

[a] MRS; deMan, Rogosa, Sharpe medium (agar)

[b] LPT + G; Lecithin Phosphate Tryptone with Glucose (agar)

Using the data gathered and equations in Appendix 2, the standard deviation, variance, and average are calculated. The standard deviations for the five conditions are pooled to yield the intermediate precision. The standard deviation for an individual plate count is calculated, followed by calculation of *SEM* for the average of three plate counts. Refer to Table 4 and section 2.4.2.1 in the manuscript for an explanation of equations and data required for calculating *SEM*.

Following is the table containing the ANOVA analysis for procedure qualification using real-life data for *L. acidophilus*. The data generated from APLM can be used to understand and control the method performance. It can also be used to compare results from other analytical procedures.

Replicate	Counts $Log_{10}CFU/g$																			
	Condition 1				Condition 2				Condition 3				Condition 4				Condition 5			
	1	2	3	Average	1	2	3	Average	1	2	3	Average	1	2	3	Average	1	2	3	Average
1	11.505	11.415	11.362	11.427	11.431	11.447	11.362	11.413	11.477	11.519	11.447	11.481	11.447	11.505	11.380	11.444	11.462	11.531	11.398	11.464
2	11.505	11.447	11.398	11.450	11.477	11.431	11.398	11.435	11.477	11.544	11.447	11.489	11.519	11.447	11.415	11.460	11.556	11.462	11.531	11.517
3	11.447	11.580	11.447	11.491	11.519	11.462	11.431	11.471	11.544	11.505	11.477	11.509	11.462	11.447	11.491	11.467	11.431	11.380	11.362	11.391
4	11.531	11.447	11.415	11.465	11.380	11.447	11.362	11.396	11.633	11.531	11.580	11.582	11.519	11.505	11.380	11.468	11.380	11.204	11.491	11.359
5	11.544	11.462	11.462	11.490	11.505	11.505	11.447	11.486	11.491	11.505	11.531	11.509	11.447	11.380	11.477	11.435	11.342	11.279	11.398	11.340
6	11.491	11.519	11.431	11.480	11.447	11.462	11.477	11.462	11.556	11.447	11.477	11.494	11.505	11.505	11.447	11.486	11.301	11.462	11.491	11.418
7	11.519	11.544	11.415	11.493	11.491	11.505	11.531	11.509	11.580	11.544	11.580	11.568	11.544	11.447	11.398	11.463	11.568	11.415	11.462	11.482
8	11.380	11.415	11.462	11.419	11.462	11.477	11.447	11.462	11.519	11.556	11.505	11.527	11.415	11.505	11.447	11.456	11.362	11.505	11.380	11.416
9	11.568	11.447	11.505	11.507	11.477	11.505	11.447	11.476	11.568	11.477	11.505	11.517	11.462	11.477	11.462	11.467	11.505	11.447	11.462	11.472
10	11.431	11.505	11.505	11.481	11.447	11.505	11.462	11.472	11.519	11.556	11.462	11.512	11.505	11.462	11.519	11.495	11.380	11.462	11.398	11.414
Std Dev (S_c)				0.02936				0.03403				0.03263				0.01771				0.05616
Variance (Sc^2)				0.0008619				0.001158				0.001064				0.0003135				0.003154
Average (C)				11.470				11.458				11.519				11.464				11.427
Intermediate Precision = Pooled Std Dev (S_{IP})				0.03620																
Std Dev for Single Plate Count (S_{P1})				0.05385																
SEM for Average of Three Plate Counts (S_{P3})				0.03109																
Std Dev for Sample Preparation (S_{PREP})				0.01854																
